# Current Status and Prospects of Pediatric Stone Disease: A Bibliometric and Visualization Study

**DOI:** 10.7759/cureus.56549

**Published:** 2024-03-20

**Authors:** Sheng Chen, Ting Zhang, Jianqiang Zhang, Xiaohan Ma, PeiZhong Wu, Shaoning Liu, Xuan Lan, Hongjun Gao

**Affiliations:** 1 Medicine, Guangxi University of Chinese Medicine, Nanning, CHN; 2 Integrative/Complementary Medicine, Ruikang Hospital, Guangxi University of Chinese Medicine, Nanning, CHN; 3 Urology, Ruikang Hospital, Guangxi University of Chinese Medicine, Nanning, CHN

**Keywords:** visualization, publications, bibliometric analysis, risk factors, pediatric stone disease

## Abstract

Pediatric stone disease, once considered rare, has emerged as a significant research area in the past two decades due to a sharp increase in its incidence. Understanding the evolving epidemiology and treatment strategies for pediatric stone disease is crucial for enhancing child health protection. This study aims to summarize the advancements in pediatric stone disease research over the last two decades through bibliometric analysis. We conducted a comprehensive search in the Web of Science Core Collection (WoSCC) for literature on pediatric stone disease from January 1, 2000 to February 20, 2024. Econometric analyses were performed using tools such as VOSviewer, CiteSpace, and the R package “bibliometrix.” Our search yielded 1,208 publications, predominantly from the United States and Turkey, showing an annual increase in publications on pediatric stone disease. Leading research institutions include Dicle University, Children's Hospital of Philadelphia, and the University of Pennsylvania, with the Journal of Pediatric Urology publishing the highest number of articles. The most prolific authors were C.P. Nelson and B. Hoppe, with Caleb P. Nelson being the most co-cited author. Research themes primarily focused on risk factors and therapeutic approaches for pediatric stone disease. Emerging research hotspots are identified by keywords such as mechanism, mini-percutaneous nephrolithotomy, recurrence, and retrograde intrarenal surgery. The study forecasts a continued upward trend in global research on pediatric stone disease, with future studies likely to delve deeper into risk factors and novel therapeutic methods.

## Introduction and background

Urolithiasis is marked by the formation or presence of mineral deposits in the urinary tract and can be further classified into nephrolithiasis, ureterolithiasis, or cystolithiasis. This condition in children, also termed pediatric stone disease, is characterized by similar mineral deposits within the urinary system [1]. The incidence of pediatric stone disease is on the rise globally. Notably, pediatric stone disease manifests differently from adult stone disease, necessitating a tailored approach to both medical and surgical treatment based on the child's metabolic condition to reduce the recurrence of surgical interventions and protect renal function [2].

The field of pediatric stone disease has seen a surge in literature during the past 20 years, but bibliometric studies have been few. This study aims to give a thorough picture of the research trends and knowledge structure in pediatric stone disease by utilizing bibliometric analysis.

## Review

Materials and methods

Data Source and Retrieval Strategies

**Figure 1:**
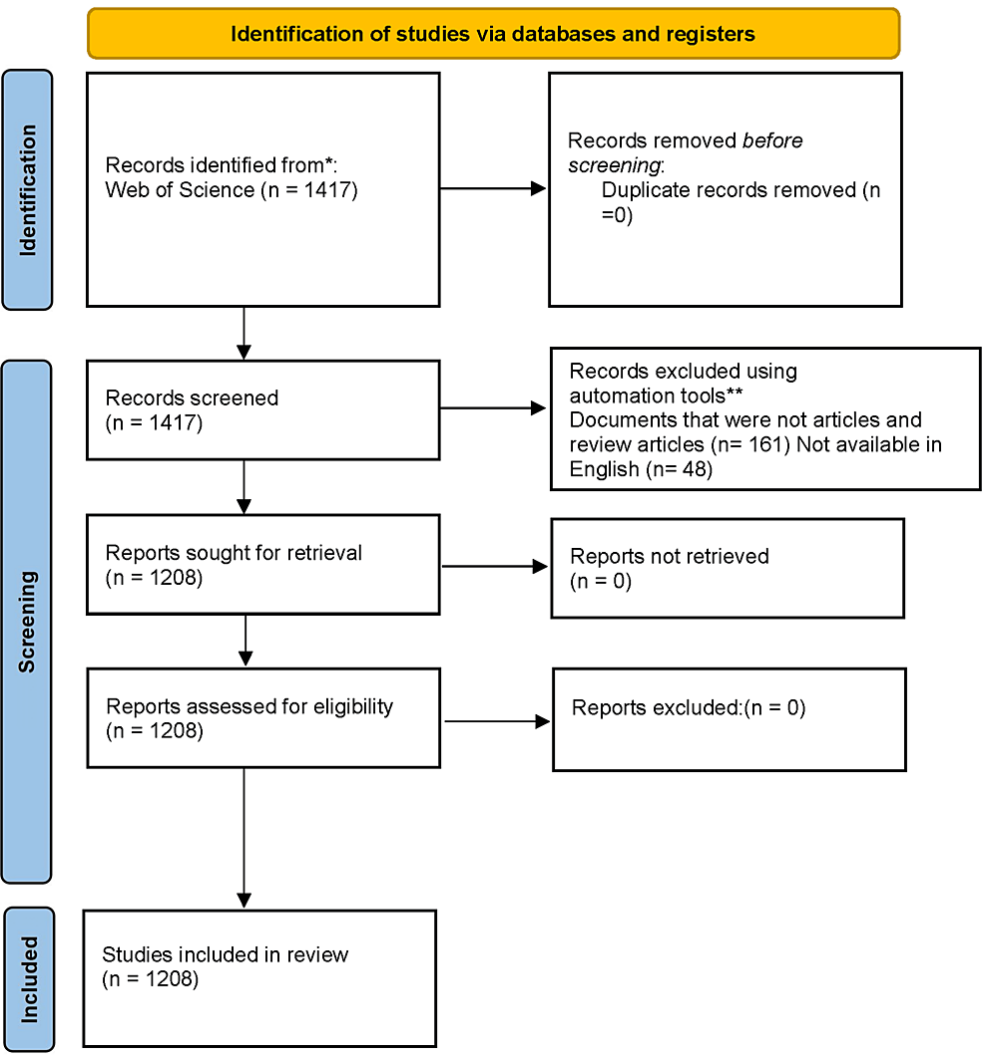
Comprehensive search flow diagram. Original image by Chen Sheng

To collect literature data, we used the Science Citation Index-Expanded database from the Web of Science. On February 29, 2024, all articles published between January 1, 2000 and February 20, 2024 were pulled from the WoSCC database and downloaded to eliminate discrepancies caused by daily database modifications. The search terms used were The search terms used were TOPICS=(Pediatric urolithiasis) OR TOPICS=(pediatric stone disease) OR TOPICS=( Pediatric nephrolithiasis) OR TOPICS=( Pediatric renal stone disease) OR TOPICS=(Pediatric urinary calculi ) OR TOPICS=(Pediatric urinary stone disease) OR TOPICS=(Pediatric renal calculi) OR TOPICS=(Pediatric renal lithiasis) OR TOPICS=(Pediatric kidney stone disease) OR TOPICS=(Pediatric urolithiasis disorder) OR TOPICS=(Pediatric nephrolithiasis syndrome) OR TOPICS=(Pediatric renal calculus disorder) OR TOPICS=(Pediatric urinary stone disorder) OR TOPICS=( Pediatric urinary lithiasis) OR TOPICS=(Pediatric urinary stone disorder). Included in the study were original research, reviews, and meta-analyses of pertinent literature on pediatric stone disease. The conference abstract themes proceedings, case report subjects, correspondence, off-topic articles, unpublished material lacking sufficient depth for further research, and duplicate papers were removed. The title, keywords, authors, references, journals, nation or area, institution, and periodicals of each article were gathered and saved as plain text files. The comprehensive WoSCC database search approach is succinctly outlined in Table 1. In the ensuing analysis, a total of 1,208 eligible publications were considered, consisting of 1,049 articles and 159 reviews (Figure 1).

**Table 1:** The search strategy of Web of WoSCC.

Research database	Web of WoSCC
Citation indexes	Science Citation Index Expanded
Query formulation	TS=(Pediatric urolithiasis) OR TS=(pediatric stone disease) OR TS=( Pediatric nephrolithiasis) OR TS=( Pediatric renal stone disease) OR TS=(Pediatric urinary calculi ) OR TS=(Pediatric urinary stone disease) OR TS=(Pediatric renal calculi) OR TS=(Pediatric renal lithiasis) OR TS=(Pediatric kidney stone disease) OR TS=(Pediatric urolithiasis disorder) OR TS=(Pediatric nephrolithiasis syndrome) OR TS=(Pediatric renal calculus disorder) OR TS=(Pediatric urinary stone disorder) OR TS=( Pediatric urinary lithiasis) OR TS=(Pediatric urinary stone disorder)
Language	English
Type of articles	Articles and Reviews
Searching period	1 January 2000 to 20 February 2024
Data collection	export with full records and cite reference in plain text format
Sample size	1208 publications including 1049 articles and 159 reviews

Data Analyses and Visualization

The WoSCC intrinsic toolkits were utilized to investigate key characteristics of the selected literature, including Web of Science subject categories, yearly publication counts, citations, and the h-index. CiteSpace is a bibliometric analysis and visualization program developed by Prof. Chen C. It can be downloaded from the website (https://citespace.podia.com/) and in our study, CiteSpace was utilized for burst word analysis [1], VOSviewer, a bibliometric analysis software that extracts key information from a large number of publications and can be downloaded from the (https://www.vosviewer.com/) website, was used in this study to assist with a number of studies, including co-occurrence analyses of institutions, authors, countries, authors, keywords, and links between scientific literature and academic hotspots [2], and Bibliometrix is an R software package containing a series of functions for quantitative scientometrics research, available from the website (https://www.bibliometrix.org/home/), which was used in this study to perform annual publication citation average analysis, national collaborative network analysis, and annual publication citation average analysis, and theme trend keyword analysis [3].

Results

Analysis of Yearly Publications and Citations

**Figure 2:**
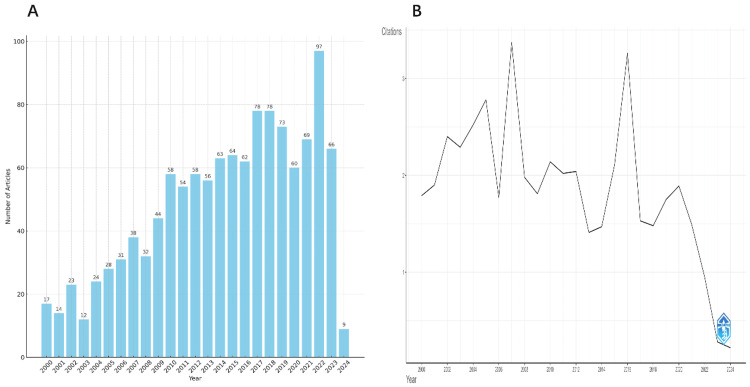
(A) Pediatric stone disease annual publications. (B) Annual publication citation average. Original image by Chen Sheng.

From WoSCC, 1,208 papers were downloaded. The yearly paper count was analyzed in EXCEL 2019. Figure 2A shows the histogram from these results. From 2000 to 2023, pediatric stone disease papers increased annually, with a more considerable rise in recent years. Since the 2008 publication, pediatric stone disease research has steadily increased, a major milestone in the area. In 2009-2023, the number of publications each year fluctuated little, while research output increased. Figure 2B shows that pediatric stone disease papers receive different numbers of citations each year. In 2007, papers averaged 3.4 citations, the most in 24 years. Articles from 2016 to 2005 average 3.3 and 2.8 citations, respectively.

Analysis of Top Countries and Institutions

**Figure 3:**
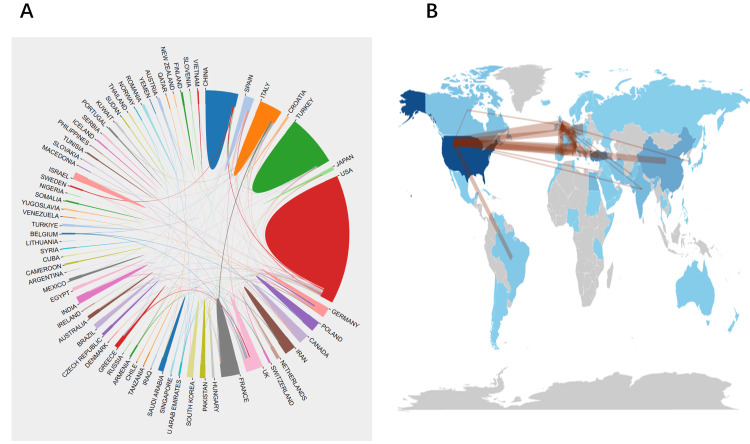
Countries' cooperative ties on pediatric stone disease (A) and the network map of collaborative relations (B). Original image by Chen Sheng.

The analysis of the cooperative network maps for pediatric stone disease-related research across nations and regions (Table 2, Figures 3A, 3B) revealed the top 10 contributing countries or regions. The United States led with 395 articles, followed by Turkey with 209, China with 103, Italy with 67, and Iran with 34. This delineation highlights geographical disparities in pediatric stone disease research output globally.

**Table 2:** Top 10 productive countries/regions in pediatric stone disease research.

Rank	Countries/Region	Publications	Link Strength
1	USA	395	63232
2	Turkey	209	56696
3	China	103	26552
4	Italy	67	19280
5	Iran	34	10397
6	India	60	19886
7	Germany	43	14908
8	France	32	5999
9	England	56	20183
10	Egypt	59	17611

**Figure 4:**
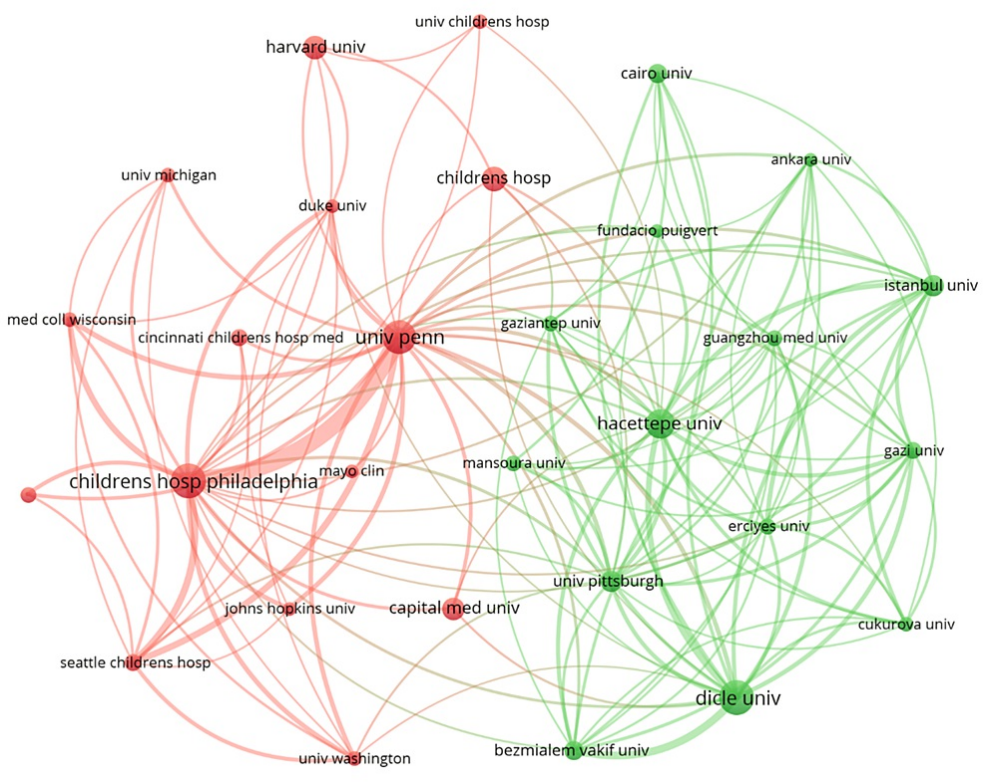
Network map of institutions. Original image by Chen Sheng.

Table 3 highlights that Dicle University, with 28 articles, Children's Hospital of Philadelphia, with 28 articles, and the University of Pennsylvania, with 27 articles, lead as the top three universities in terms of pediatric stone disease research contributions. A closer look at the collaboration connection strength and the institutional network collaboration map (Figure 4) reveals the potential for amplifying global cross-institutional collaboration in pediatric stone disease research. This observation suggests that while certain institutions are pioneering in the field, there exists an opportunity to further global collaborative efforts to enhance research outcomes and innovation in information technology.

**Table 3:** Top 10 productive institutions in pediatric stone disease research.

Rank	Institutions	Publications	link strength
1	Dicle University	28	2758
2	Children's Hospital of Philadelphia	28	3330
3	University of Pennsylvania	27	3957
4	Hacettepe University	23	2378
5	Harvard University	19	799
6	Capital Medical University	18	1072
7	University of Pittsburgh	17	2006
8	Istanbul University	17	1712
9	Cairo University	15	979
10	Bezmialem Vakif University	15	1976

H-index of Authors and Referenced Authors

**Figure 5:**
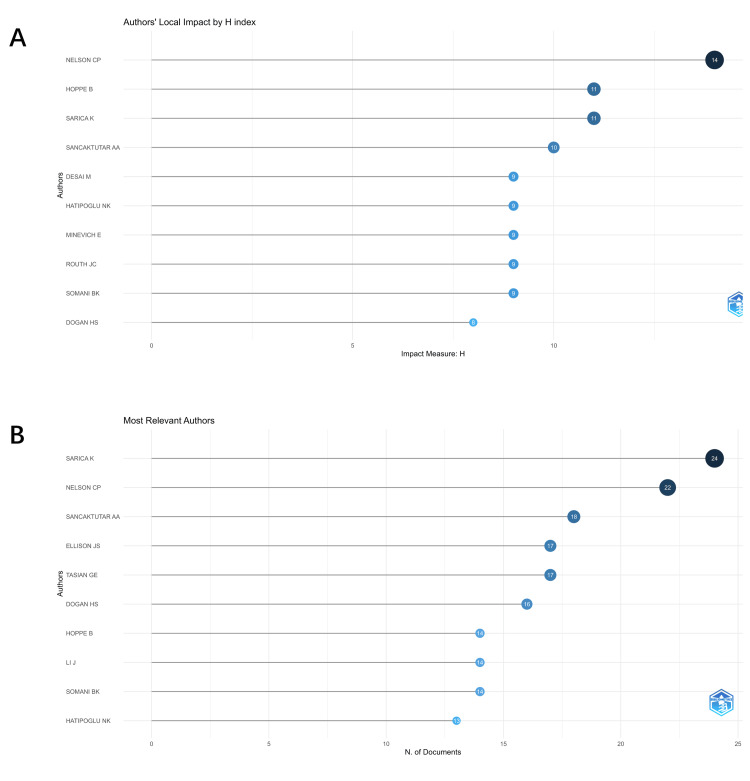
(A) Top 10 pediatric stone disease domain author H index. (B) Author article count. Original image by Chen Sheng.

Authorship is a key sign of dissertation productivity and helps advance the field. Current pediatric stone disease academics were identified by assessing their publication production and prospective linkages. Figure 5A shows writers with pediatric stone disease publications' top 10 local impact H-indexes.

The leading author in the field is C.P. Nelson, with 14 publications, while B. Hoppe and K. Sarica are tied for second place, each with 11 publications. As illustrated in Figure 5B, the top two authors with the highest H-index are also among those with the most articles published. Although M. Desai ranks fifth in terms of the H-index, he does not appear among the top 10 authors by publication count, attributing to a relatively smaller number of publications. Notably, E. Minevich, despite not being listed in the top 10 for the number of published articles, is recognized for the high quality of his contributions.

**Figure 6:**
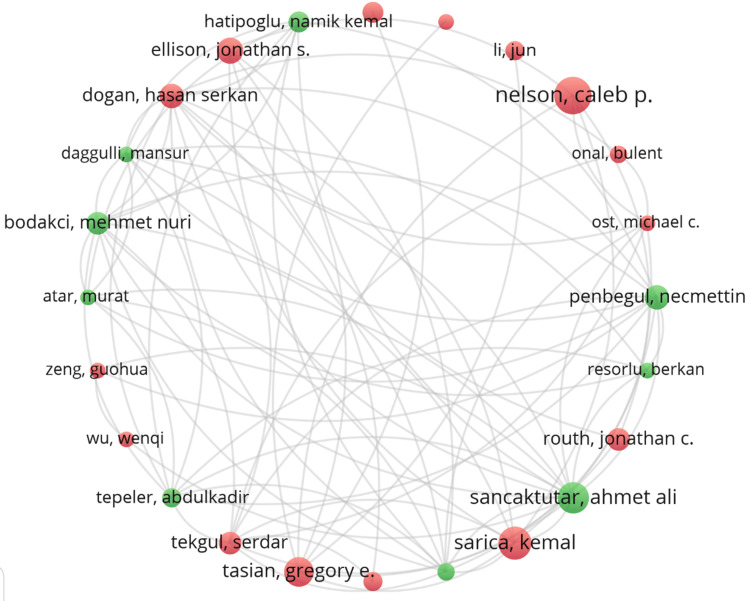
Cooperation map of authors. Original image by Chen Sheng.

Figure 6 shows the authors' collaborative network's co-occurrence diagram, where colored circles represent clusters and lines indicate cooperation strength. The top five writers with the most publications are listed in Table 4. Caleb P. Nelson (22 articles), Kemal Sarica (19 articles), Ahmet Ali Sancaktutar (18 articles), Gregory E. Tasian (17 articles), and Jonathan S. Ellison (15 articles). 15 articles). Co-cited authors are also shown, with the most co-cited authors being Caleb P. Nelson (1,703 co-citations), Kemal Sarica (797 co-citations), Jonathan C. Routh (418 co-citations), Gregory E. Tasian (255 co-citations) and Ahmet Ali Sancaktutar (254 co-citations). These authors have had a significant scholarly impact in the field of pediatric stone disease.

**Table 4:** The top 10 productive and cited authors on the research of pediatric stone disease.

Rank	Author	Documents	Co-cited author	Count
1	Caleb P. Nelson	22	Caleb P. Nelson	1703
2	Kemal Sarica	19	Kemal Sarica	797
3	Ahmet Ali Sancaktutar	18	Jonathan C. Routh	418
4	Gregory E. Tasian	17	Gregory E. Tasian	255
5	Jonathan S. Ellison	15	Ahmet Ali Sancaktutar	254
6	Hasan Serkan Dogan	14	Necmettin Penbegul	234
7	Necmettin Penbegul	14	Hasan Serkan Dogan	191
8	Mehmet Nuri Bodakci	13	Mehmet Nuri Bodakci	182
9	Jonathan C. Routh	13	Jonathan S. Ellison	164
10	Serdar Tekgul	13	Serdar Tekgul	160

Analysis of Journals

**Figure 7:**
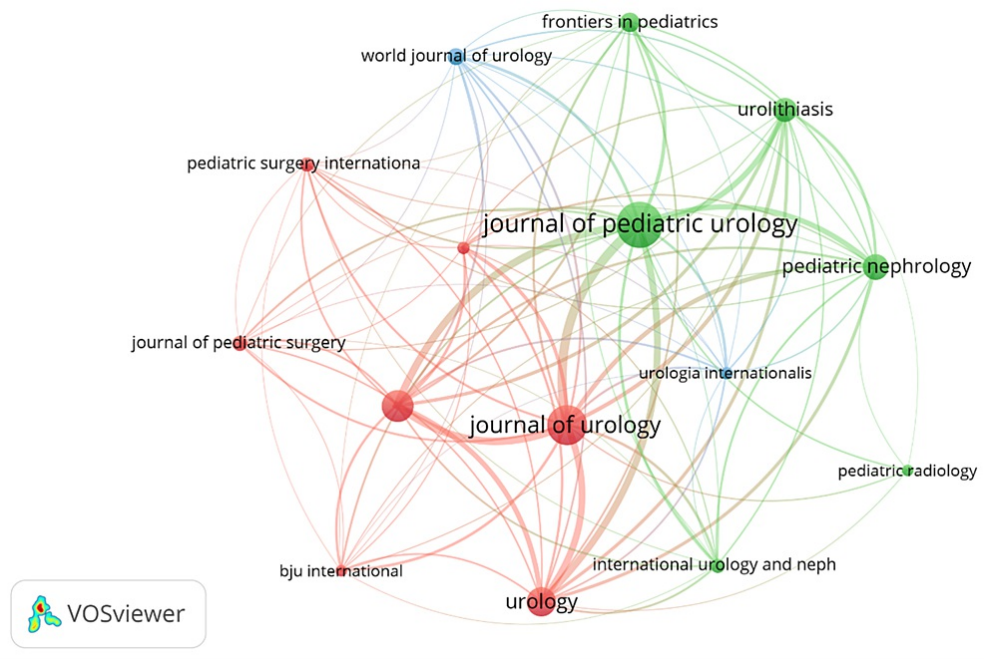
Cooperation map of journals. Original image by Chen Sheng.

From January 1, 2000 to February 20, 2024, a total of 1,208 research papers related to pediatric stone disease were published in 265 journals, of which 21 journals published at least 10 articles. We analyzed the journals and created a network diagram (Figure 7). Table 5 shows the top 10 field journals by publication. Journal of Urology has the highest IF of 6.60 among the top 10 journals. In addition, the World journal of Urology (2022 IF:3.40), Urolithiasis (2022 IF:3.10), and Pediatric Nephrology (2022 IF:3.00), all of which have published more than 20 articles. Combining the Journal Citation Reports (JCR) evaluation system's data, we also observed that the top 10 journals were mostly concentrated in Q1 and Q2, indicating a strong impact in the associated sectors. In Table 6, the Journal of Urology has the most citations with 6627, followed by the Journal of Endourology with 2,041 and Pediatric Nephrology with 1,822.

**Table 5:** Top 10 most-articled journals.

Rank	Source	2022 IF	JCR	Publications	Citations
1	Journal of pediatric urology	2.00	Q3	141	1479
2	Journal of urology	6.60	Q1	113	6627
3	Journal of endourology	2.70	Q2	76	2041
4	Urology	2.10	Q3	67	1465
5	Pediatric nephrology	3.00	Q2	55	1822
6	Urolithiasis	3.10	Q2	47	405
7	Frontiers in pediatrics	2.60	Q2	35	167
8	World journal of urology	3.40	Q2	27	483
9	Journal of pediatric surgery	2.40	Q2	22	428
10	International urology and nephrology	2.00	Q4	21	298

**Table 6:** Top 10 most-cited journals.

Rank	Source	2022 IF	JCR	Citations
1	Journal of urology	6.60	Q1	6627
2	Journal of endourology	2.70	Q2	2041
3	Pediatric nephrology	3.00	Q2	1822
4	Journal of pediatric urology	2.00	Q3	1479
5	Urology	2.10	Q3	1465
6	BJU international	4.50	Q1	489
7	World journal of urology	3.40	Q2	483
8	Journal of pediatric surgery	2.40	Q2	428
9	Urolithiasis	3.10	Q2	405
10	Kidney international	19.60	Q1	395

Analysis of Keywords

**Figure 8:**
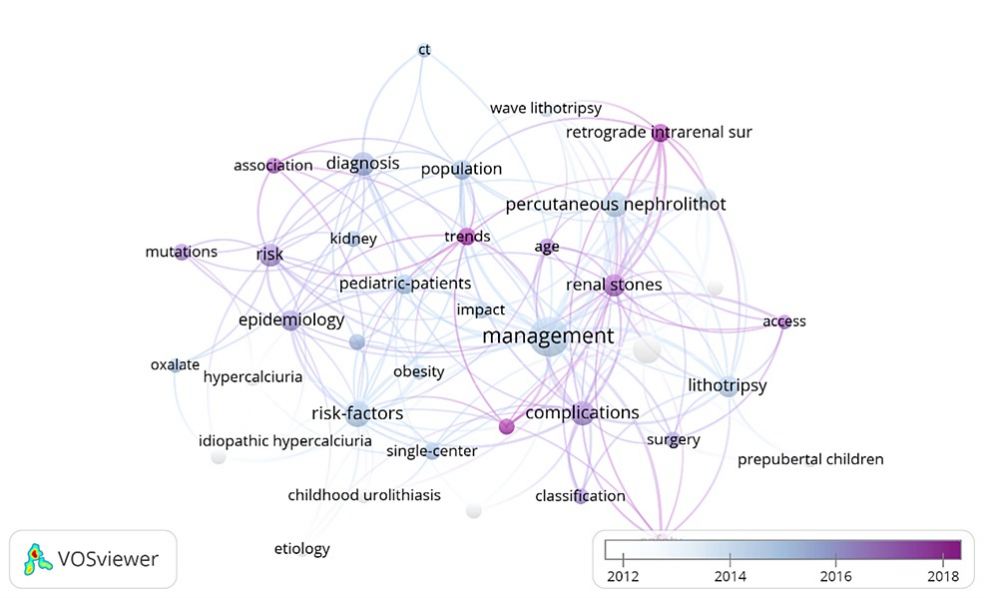
Keyword co-occurrence network. Original image by Chen Sheng.

The important points of a piece of literature are its keywords, and keyword co-occurrence analysis might reveal research hotspots. VOSviewer visualizes the 40 keywords with a frequency greater than or equal to 22. The larger the node, the higher the keyword frequency and research hotness; the thicker the line between the nodes, the more the keywords are used in the literature. Figure 8 shows that the 40 nodes are research hotspots in this subject and have a close link.

**Figure 9:**
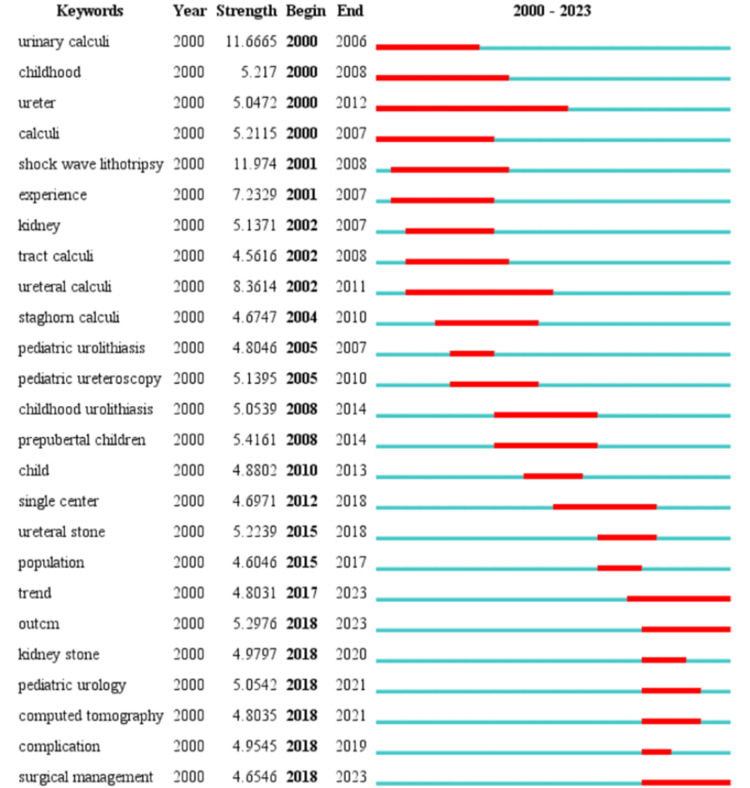
Top 25 Keywords with the strongest citation bursts. Original image by Chen Sheng.

In order to gain further insight into the sudden emergence of hot research in the field, the choice was made to use CiteSpace's burst word analysis feature and the resulting data is shown in Figure 9. In this study, the burst words explored aspects of treatment, diagnosis, and risk factors for pediatric stone disease.

**Figure 10:**
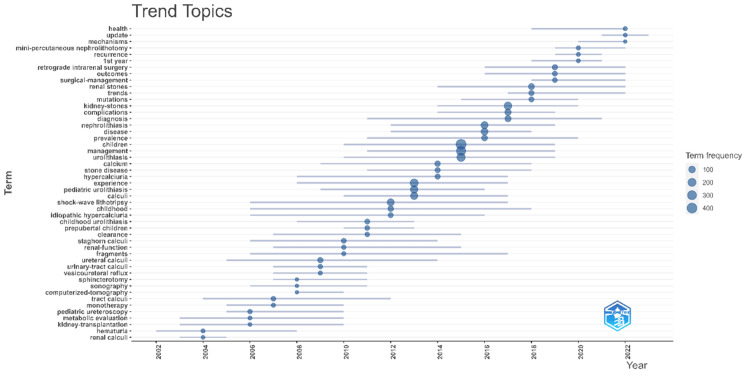
Theme trend keyword. Original image by Chen Sheng.

Figure 10 shows the trend chart of the topic words, one can find in this chart mechanism, mini-percutaneous nephrolithotomy (PCNL), recurrence, retrieval intrarenal surgery, outcomes, surgical-management, and other trending words are identified as the latest trending topics.

Discussion

The research hot spots and development trajectory in the field of pediatric stone disease are completely summarized by incorporating the keyword co-occurrence map, burst word map, and theme trend keyword map.

Risk Factors of Pediatric Stone Disease

Various factors contribute to the formation of kidney stones, encompassing the concentration of stone-forming ions in the urine, the urine's pH and flow rate, factors influencing metabolic crystallization, and anatomical considerations [4-8]. European pediatric studies initially indicated infection as the primary cause of urolithiasis [9,10]. However, recent trends show metabolic factors now play a dominant role in pediatric urolithiasis cases [11-13]. Indeed, metabolic issues are implicated in the majority of pediatric cases with stone disease [7,8,12-14].

**Figure 11:**
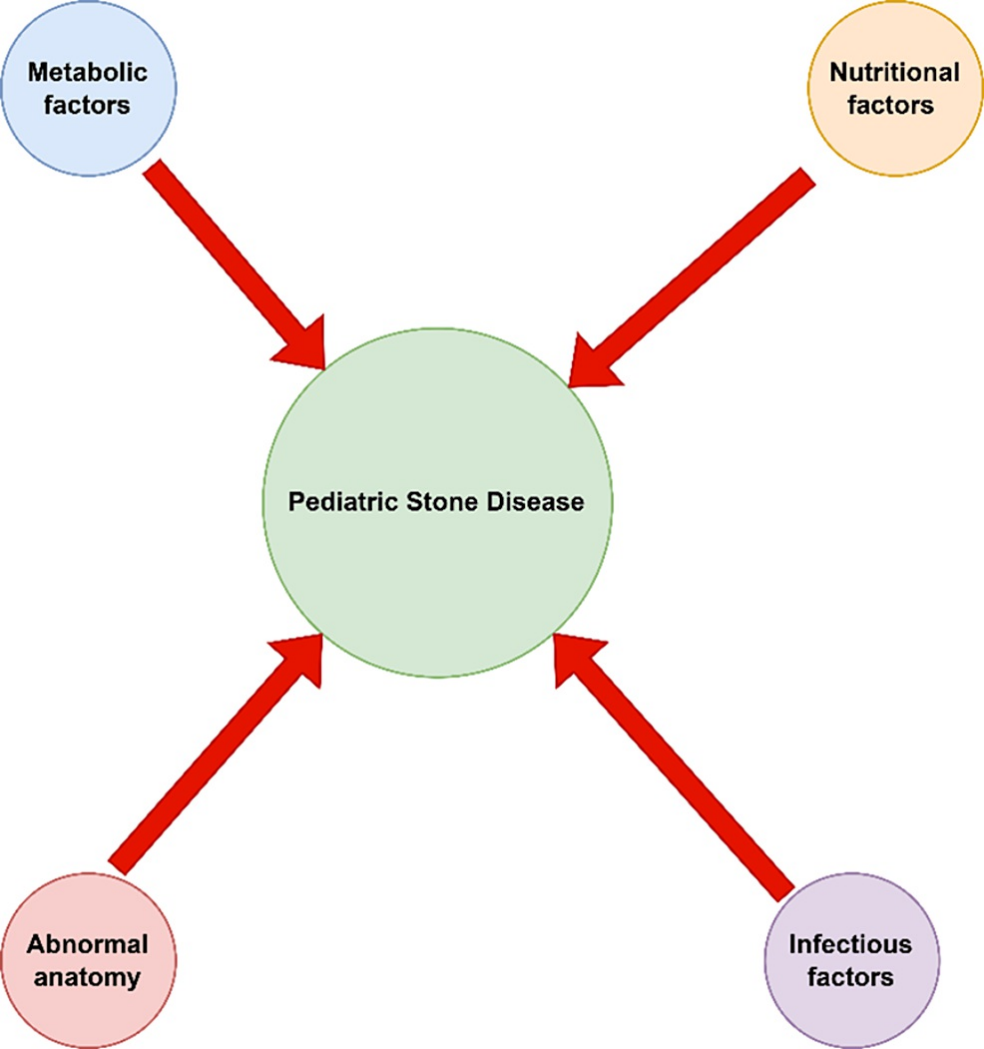
Risk factors of pediatric stone disease. Original image by Chen Sheng.

Hypercalciuria stands as a pivotal metabolic risk factor for pediatric stone disease [15]. Notably, this condition may precipitate recurrent urinary tract infections and a notable reduction in children's bone mineral density [7,16]. Concurrently, hyperoxaluria emerges as another critical risk factor for urinary stones in this demographic [17,18]. Anatomical abnormalities in the urinary tract, like ureterocele, substantially increase the risk of stone formation. This is because they contribute to urinary retention, which can lead to infections that further exacerbate the risk [17-19]. Nutritional decisions can also greatly impact a child's stone risk. A protein-rich diet leads to an increase in uric acid, calcium, and oxalate, thereby increasing the risk of stone formation [20]. Additionally, the intake of oxalate-rich foods, including turnips, strawberries, chocolate, parsley, beets, and nuts, is known to trigger hyperuricemia [13]. The role of diet extends to specific dietary patterns, such as the ketogenic diet, and broader lifestyle factors like a high-fat diet and obesity, both of which are associated with an increased stone risk [21,22]. Risk factors of pediatric stone disease, are summarized in Figure 11.

Treatment of Pediatric Stone Disease

Surgical intervention becomes necessary for children when ureteral stones cannot pass on their own or when kidney stones start showing symptoms. Surgery is required in up to 22% of acute pediatric stone episodes [23]. Shock Wave Lithotripsy (SWL) can treat pediatric stone disease [24]. SWL offers the benefits of being less invasive with a high stone clearance rate [25]. However, potential complications include intestinal perforation, renal colic, and hematuria [26-28].

Recent analysis shows an increase in Retrograde intrarenal surgery (URS) utilization [29], with significant advancements in techniques and technology [30,31]. URS uses a ureteroscope to treat ureteral and kidney stones retrogradely, clearing big stones [32]. The American Urological Association recommends URS for pediatric patients unlikely to pass stones naturally or who have not responded to other treatments [33]. URS has become the preferred method for treating renal stones smaller than 20 mm in pediatric patients, with stone-free rates of 95% for stones smaller than 10 mm and 78% for larger stones [34-36]. A prospective comparative study between URS and SWL for 10-20 mm stones in 60 children showed stone clearance rates of 93.3% and 96.6%, respectively, without serious adverse effects [37]. However, URS can lead to complications such as ureteral perforation, urinary tract infections, hematuria, pain, and bleeding [38].

**Figure 12:**
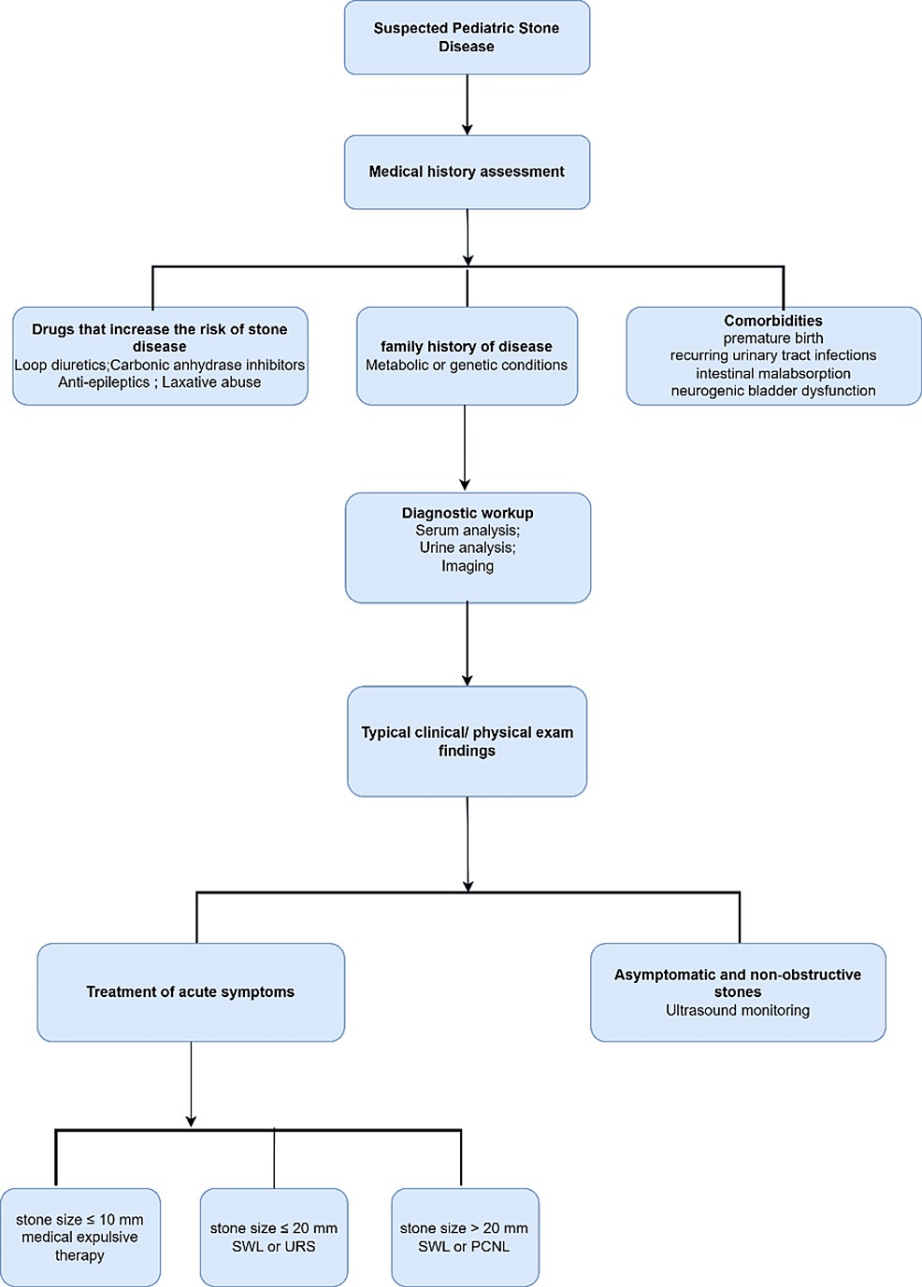
Diagnosis and treatment process of pediatric stone disease. Original image by Chen Sheng.

PCNL, was introduced for pediatric patients in 1985 [39]. It is the most invasive option, requiring general anesthesia to insert a needle into the kidney's collecting system, expand the access channel, and use nephroscopes for stone removal [40]. Preoperative management includes evaluating and treating urinary tract infections, with antibiotics administered perioperatively [41]. The American Urological Association suggests PCNL for kidney stone burdens greater than 20 mm, citing a stone clearance rate of over 90% [33]. Complications can include bleeding, organ damage, and incomplete stone removal [42,43]. Figure 12 outlines the diagnostic and treatment process for pediatric kidney stones, following American Urological Association guidelines [33,44].

## Conclusions

Recent years have seen a rise in pediatric stone disease research articles, which has greatly contributed to the development of this field. However, collaboration between academia has yet to be prompted. Research collaborations between different regions and universities can be strengthened in the future. Current research focuses on the treatment and risk factors of pediatric stone disease. Researchers can further improve high-quality clinical research, broaden the scope of research, and provide better strategies for the early diagnosis and treatment of pediatric stone disease.
